# Transfusion from Lamb’s Blood

**Published:** 1874-09

**Authors:** 


TRANSFUSION FROM LAMB'S BLOOD.—* * * The effect of the operation was almost nothing. Regarding the phthisical patients, neither sleep nor appetite, nor any local symptom, showed the slightest alteration. The weight of their bodies within the first five or six weeks after the injection of blood, decreased in the same proportion as before; the same result was observed in all the other cases. One patient (puerperal disease) died two days after the operation, but the autopsy afforded no reason to believe that the fatal end was due to the therapeutic measure.

It seems, therefore, that the wonderful tales related by Gesellius are not quite trustworthy, and that, if transfusion should be necessary as *ultimum refugium*, nothing but human blood ought to be used for the purpose.—*London Lancet.*

